# COVID-19 vaccination-triggered cluster headache episodes with frequent attacks

**DOI:** 10.1177/03331024221113207

**Published:** 2022-07-13

**Authors:** Roemer B Brandt, Rosa-Lin H Ouwehand, Michel D Ferrari, Joost Haan, Rolf Fronczek

**Affiliations:** 1 Department of Neurology, Leiden University Medical Centre (LUMC), Leiden, The Netherlands; 2 Department of Neurology, Alrijne Hospital, Leiderdorp, The Netherlands

**Keywords:** Cluster headache, COVID-19 vaccination, headache

## Abstract

**Background:**

The pathophysiology of cluster headache and how cluster episodes are triggered, are still poorly understood. Recurrent inflammation of the trigeminovascular system has been hypothesized. It was noted that some long-term attack-free cluster headache patients suddenly developed a new cluster episode shortly after COVID-19 vaccination.

**Methods:**

Cases are described from patients with cluster headache who reported a new cluster episode within days after COVID-19 vaccination. All cases were seen in a tertiary university referral center and a general hospital in the Netherlands between March 2021 and December 2021, when the first COVID-19 vaccinations were carried out in The Netherlands. Clinical characteristics of the previous and new cluster episodes, and time between the onset of a new cluster episode and a previous COVID-19 vaccination were reported.

**Results:**

We report seven patients with cluster headache, who had been attack-free for a long time, in whom a new cluster episode occurred within a few days after a COVID-19 vaccination.

**Interpretation:**

COVID-19 vaccinations may trigger new cluster episodes in patients with cluster headache, possibly by activating a pro-inflammatory state of the trigeminocervical complex. COVID-19 vaccinations may also exacerbate other neuroinflammatory conditions.

## Introduction

Activation of the trigeminocervical complex, causing the release of calcitonin gene related peptide (CGRP) and other neuropeptides, and inflammation of the trigeminovascular system and cavernous sinus have been implicated in the pathophysiology of cluster headache (1). CGRP is a potent vasodilator and neurotransmitter and can induce a pro-inflammatory state through the release of nitric oxide (NO) and activation of satellite glial cells and mast cell degranulation (2). Individual attacks can be triggered by alcohol, CGRP and NO, but only in people with chronic cluster headache (CH) or in those with episodic CH who are in an active episode (3,4). Attacks cannot be triggered in attack-free periods or in people without CH. Apart from the fact that some patients suffer more often from cluster episodes in spring than in other seasons, we know of no other proven trigger for cluster episodes (5,6).

Since the start of COVID-19 vaccination in the Netherlands in January 2021, several long-term attack-free CH patients reported the onset of a new unexpected cluster episode shortly after vaccination. Here we report seven such cases suggesting that COVID-19 vaccination may trigger a cluster episode in patients with a history of CH. The study was approved by the local ethics committee of the LUMC and all patients provided informed consent (METC LDD; protocol number G21.055).

## Case reports

The cases are summarized in Table 1 and presented below.

**Table 1.** Patient characteristics.

Case #	Age	Sex	CH type	Usual attack frequency	‘Normal’ AI (NRS)	Vaccine	Onset (days after vaccine)	Duration of exacerbation	Daily AF after vaccine	AI after vaccine (NRS)	Reason vaccination is suspected as cause	Atypical feature(s)
1	50s	Male	Episodic	1–6/day	10	Ad26.COV2-S 1st vaccination	5	>6 months	1–6/day	10	Second episode in short succession (normally 1–1,5 years in between)	Longer duration (normally 6–8 weeks), verapamil and GON injection ineffective
2	60s	Male	Episodic	2–3/day	8–10	ChAdOx1-S1st vaccination	1	>4 months	2–8/day	8–9	Prolonged period (3.5 years) of attack freedom	Attack intensity and frequency appears higher
3	40s	Female	Episodic	2–3/day	10	BNT162b2 2nd vaccination	3	3 months	2/day	6–8	Earlier start than usual (mid-July instead of end of August)	None
4	60s	Male	Episodic	2–10/day	10	ChAdOx1-S1st vaccination	2	3 weeks	3/day	6	Temporal relation and atypical duration	Lower attack intensity, shorter episode duration (normally 3–6 months)
5	50s	Male	Chronic	1–8/day	9–10	ChAdOx1-S1st and 2nd vaccination	0 (20/5 minutes)	3 weeks (both)	4–5/day	8	Attacks appeared after a prolonged period of attack freedom (6 years)	6-year attack freedom from previous chronic CH
6	70s	Female	Chronic	3–4/day	10	BNT162b2 2nd vaccination	1	≫5 months	3–4/day	10	Prolonged period of attack freedom (7 years), only ‘shadow attacks’	None
7	70s	Male	Episodic	3/day	10	BNT162b2 3rd vaccination	7	>1 month	8/day	7	Prolonged period of attack freedom.	Higher attack frequency and lower attack intensity, GON injection ineffective

AF, attack frequency; AI, attack intensity; CH, cluster headache; GON, greater occipital nerve.

### Case 1

A man in his mid-50s had regular episodes of CH every 1 to 1.5 years between 1999 and March 2021. Five days after vaccination with Ad26.COV2-S in May 2021, he unexpectedly had another episode. Unlike previously, verapamil and an injection of the greater occipital nerve (GON) with corticosteroids and lidocaine were not effective. The episode ended after six months after a second GON-injection.

### Case 2

This man in his 60s with chronic CH had been attack-free with verapamil 360 mg/day for 3.5 years. One day after his first vaccination with ChAdOx1-S, the attacks returned, with a higher pain intensity than before. The attacks disappeared briefly during a 60-day course of high dose prednisone and he was attack-free after increasing the verapamil dose to 480 mg daily. However, mild ‘shadow headaches’ with tearing of the eye continued once or twice a week.

### Case 3

A woman in her mid-40s had an episode of CH for 3–4 months every year, always starting in August and in February. In early July, three days after her second vaccination with BNT162b2, a new episode started for 3 months. In contrast with previous episodes, a GON-injection and verapamil were not effective in this episode.

### Case 4

This man in his 60s had episodes of CH that usually lasted 3–9 months. A new episode started two days after his first vaccination with ChAdOx1-S. The attacks felt the same as before but the pain was less severe. Unlike previous episodes, this episode lasted only three weeks.

### Case 5

A man in his mid-50s had chronic CH until six years ago. Twenty minutes after his first vaccination with ChAdOx1-S and five minutes after his second vaccination, he had new episodes of CH, each lasting three weeks and with an attack-frequency of 4–5 per day.

### Case 6

This woman in her 70s with chronic CH had been attack-free with lithium carbonate 2 × 300 mg (serum level 0.83 mmol/L) since 2014. She just had some ‘shadow-attacks’ every month. In June 2021, one day after a second vaccination with BNT162b2, the attacks of CH reappeared up to four times a day. She increased the lithium dose herself to 1200 mg per day, which resulted in lithium intoxication and admission to the intensive care and dialysis. After discharge she remained attack free without medication.

### Case 7

This man in his 70s had episodic CH since 2000, permanent miosis since 2012, and CCH since 2014 with three attacks per day. After variable success of treatment with verapamil, he became attack-free after GON injections in June 2020 and January 2021 until December 2021 when he received a booster vaccination with BNT162b2. Within a week, the attacks returned to 8 per day. A repeat GON injection had no decisive effect. Interestingly, he used to have an exacerbation of his CH after every influenza vaccination and had stopped doing so in 2015.

## Discussion

We report seven patients with CH who, after having been attack-free for a long time, suddenly developed a new cluster episode shortly after COVID-19 vaccination with frequent attacks for several weeks to months. A correlation in time may, of course, be coincidental. However, in all seven patients the cluster episodes occurred just after vaccination after prolonged attack freedom, and/or occurred in different parts of the year than would normally be the case. In one of these patients, the cluster episodes occurred immediately after both the first and second vaccination.

These observations suggest that both vector based (Ad26.COV2.S, ChAdOx1) and RNA vaccines (BNT162b2) against COVID-19 may elicit cluster episodes in CH patients. Although transient headaches are a common complication of COVID-19 vaccination (7,8), particularly in those with migraine or tension-type headache, we are not aware of any other reports of COVID-19 vaccination induced cluster episodes (9,10). Based on a prevalence of CH of ±0.1% (1) and a vaccination rate of >85% in the Netherlands, one would expect more cases, but underreporting is not unlikely in this rare and often unrecognized condition.

The occurrence of “shadow attacks”, with mild or incomplete symptomatology, during remission periods and especially just before a subsequent cluster episode (11), suggests that the underlying disease activity in people with CH fluctuates, even during attack-free periods in people with chronic CH (3,6). Cluster episodes probably occur only when the disease activity exceeds a certain threshold (11,12).

It is hypothesized that in people with CH, the trigeminovascular system is in a constant fluctuating pro-inflammatory state (11,12) and that certain unknown events cause trigeminal activation, CGRP release and trigeminovascular inflammation (1). High doses of prednisone, a potent anti-inflammatory and CGRP release-inhibiting drug (13), may indeed effectively block the occurrence of CH attacks (1). Many of COVID-19 triggered headaches are migraine-like, which also seems to indicate neuroinflammation (2) and trigeminal activation with consequent release of CGRP (7,9). Vaccination evokes an antibody and cellular immune response that could promote the transition from pro-inflammatory to full inflammation. Similarly, an increased autoimmune response induced by COVID-19 vaccination via antigenic cross-reactivity between the spike protein of the SARS-CoV-2 and self-antigens, may cause an increase in the relapse rate of multiple sclerosis (14,15). Alternatively, a direct central (inflammatory) role of the vaccine could play a role in the occurrence of a cluster episode as well, with a recent case report describing a (transient) loss of blood-brain barrier integrity shortly after a second dose of COVID-19 vaccine (16).

We can only speculate why we seem to observe a cluster episode following only vector and mRNA based COVID-19 vaccinations, and apparently not from vector or other vaccinations against other diseases. It is possible that COVID-19 vaccinations exert a stronger immunological response or that there is an antigenic cross-reactivity between the spike protein of the SARS-CoV-2 and self-antigens in some people that is not observed in other vaccines. This would imply that the COVID-19 virus itself could possibly trigger a cluster episode as well, although we did not observe this phenomenon, possibly because it is still relatively early in the pandemic. Finally, it is an option that the association was only noticed because extremely large numbers of people were vaccinated in a very short period of time and there was a great deal of focus on possible complications. Remarkably, one patient reported that influenza vaccinations in the past seemed to provoke cluster episodes in him as well.

Some patients reported some atypical features of their episode (e.g. longer episode duration, not responsive to their typical medication). This could be due to an atypical trigger of the cluster episode which could possibly cause a slightly different phenotype of their episode. However, the attacks within their episode were evidently cluster headache attacks. Furthermore, none of the patients reported any changes in response to attack medication.

## Conclusion

COVID-19 vaccination may elicit cluster episodes reinforcing a potentially important pathophysiological role of inflammation of the trigeminocervical complex in CH.

## Article highlights

- COVID-19 vaccination could be a trigger for cluster headache episodes.
- This trigger could imply an important role for neuroinflammation in the pathophysiology of cluster headache.
